# From anecdotes to evidence: Environmental DNA detection of Arctic charr (*Salvelinus alpinus* L.) at the southern limit of its circumpolar range

**DOI:** 10.1111/jfb.16048

**Published:** 2025-01-22

**Authors:** Molly Ann Williams, Samuel J. Poultney, Jane Hallam, Tianna E. Hewitson, Joanne E. Littlefair

**Affiliations:** ^1^ NatureMetrics, Guildford Surrey UK; ^2^ School of Life Sciences University of Warwick Coventry UK; ^3^ Dee District Salmon Fishery Board, Aboyne Aberdeenshire UK; ^4^ West Sutherland Fisheries Trust, Sutherland UK; ^5^ Yale School of the Environment Yale University New Haven Connecticut USA; ^6^ School of Biological and Behavioural Sciences Queen Mary University of London London UK; ^7^ Department of Genetics, Evolution and Environment University College London London UK

**Keywords:** Arctic charr, climate change, eDNA, freshwater monitoring, Scotland

## Abstract

The urgency of rapid species monitoring is at an all‐time high due to the increasing threat of climate change to global ecosystems, in particular freshwater habitats. Fish such as Arctic charr, *Salvelinus alpinus*, are particularly vulnerable to increasing water temperatures and changes in land use due to their dependence on cold waters and confinement to lacustrine environments. Nonetheless, current monitoring practices, relying on physical capture of organisms, are hindered by resource constraints, desire to manage habitats for recreational fishing, and restricted access to sites. Here we applied a targeted environmental DNA (eDNA) assay in Northwest Scotland to circumvent these limitations and update existing knowledge of Arctic charr habitats, including in locations previously only supported by anecdotal knowledge. Arctic charr eDNA was detected in 10 out of the 16 sites sampled. Additionally, shore and outflow sampling successfully detected Arctic charr eDNA during spawning season, providing a viable sampling strategy where boat access may be limited. These data enabled Arctic charr distribution records to be updated and demonstrated the effectiveness of eDNA as a method for monitoring a vulnerable salmonid in a rapidly changing landscape.

## 1 INTRODUCTION

Monitoring of freshwater fishes across Great Britain is vital to implement protections in the face of climate change. Scotland contains 90% by volume of the standing fresh waters of Great Britain (Lyle & Smith, 1994), and 4 years of monitoring data has demonstrated that 97% of Scottish lochs and reservoirs experienced warming between 2015 and 2019, with the majority warming by 0.25 to 1.0°C per year (May et al., 2022). Although this is a short‐term dataset, the overall trend is expected to continue with an average of 3°C of warming predicted for Scottish lochs by 2080 (May et al., 2022). Additionally, climate models predict an increase in the duration and intensity of extreme short‐term lake heat waves, which will produce short‐term but dramatic fluctuations above average temperatures (May et al., 2022). The Scottish Environment Protection Agency (SEPA) is projecting an increase in drought events from 1 in 20 to 1 in 2 years (SEPA, 2024). Such heating in Scottish lakes will have a profound effect on stenothermic (cold‐water) freshwater fishes inhabiting the lochs, including native *Salvelinus alpinus* L., Arctic charr.

Arctic charr is a freshwater fish species in Scotland (Maitland & Adams, 2018), with the wider species range exhibiting a Holarctic distribution (Klemetsen et al., 2003). They naturally occur in 16 Northern Hemisphere countries, including Britain (Frost, 1963; Maitland, 1995; Maitland et al., 2007; Winfield et al., 2010) and Ireland (Maitland, 1995; Maitland et al., 2007; Winfield et al., 2010). Climate change is the overriding international and national factor affecting Arctic charr, especially at the southern limits of its distribution (Arthington et al., 2016; Winfield et al., 2010; Winfield et al., 2016). Arctic charr are habitat generalists, with lacustrine populations being the most common, using all major habitat and depth zones within lochs (Klemetsen et al., 2003). As a stenothermic fish, they have an optimum temperature for growth between 12 and 17°C depending on population of origin, environmental factors, and size of individual (Beuvard et al., 2022; Imsland et al., 2020; Jobling, 1983; Jobling et al., 1993; Larsson et al., 2005; Larsson & Berglund, 2005), with 50% of juveniles assayed able to survive up to 19–22°C (Baroudy & Elliott, 1994). Consequently, Arctic charr are the UK's most vulnerable salmonid to observed and predicted temperature change, as non‐anadromous populations are unable to disperse through marine habitats but are instead restricted by discrete freshwater lochs (IPCC, 2018; Kendon et al., 2019; Lehnherr et al., 2018). Furthermore, suitable habitat for Arctic charr is reduced by changing land and water usage, which degrades water quality and alters hydrological regimes (Klemetsen et al., 2003; Maitland, 1995; Maitland et al., 2007). Additionally, fish introductions can lead to competition for resources and predation, further threatening native Arctic charr populations (Corrigan et al., 2011; Winfield et al., 2011, 2012).

The foundation for our work is a Scottish‐centric index and review of historical and current data on Arctic charr lochs, called for 130 years ago (Maitland & Adams, 2018). There are approximately 30,000 lochs in Scotland, but only 295 freshwater sites with informative records on Arctic charr. In Northwestern Scotland particularly, there is a data deficiency for lochs that appear superficially suitable for Arctic charr (Ferguson et al., 2019; Maitland & Adams, 2018). This under‐recording of Arctic charr presence is primarily due to the limited commercial or recreational exploitation of their populations, as angling interest focuses on Atlantic salmon (*Salmo salar* L.) and brown trout (*Salmo trutta* L.). Increased and continued mapping of this vulnerable salmonid will better characterize their presence across Scotland, allowing predictions to be made about the responses of Arctic charr populations to future increases in water temperatures.

Increasingly, scientists and habitat managers are turning to new technologies to gather rapid, comprehensive data on vulnerable populations where environmental stressors are likely to be accelerating. For example, AI classification of images, passive bioacoustics, remote sensing, and genetic technologies are being established to increase the spatial, temporal, and taxonomic scales on which we can understand biodiversity. Environmental DNA (eDNA) is enhancing our ability to monitor freshwater fish species in a relatively quick, cost‐effective, and non‐invasive manner (Rees et al., 2014). Targeted approaches such as quantitative PCR (qPCR) are of particular use due to their high sensitivity compared to broader community analysis (Yu et al., 2022). These methodologies are useful when assessing fish stocks in areas where existing evidence may be poor, such as in regions with outdated records, lack of formal studies, or anecdotal species presence only. This is the case for the presence of Arctic charr in many of Scotland's lochs. Additionally, the non‐invasiveness of sampling within eDNA studies allows monitoring to be conducted where traditional survey methods, such as gillnetting, may be opposed by land managers due to the lethal effect on sports fish.

Previous studies have shown that Arctic charr are amenable to monitoring using targeted eDNA assays (Doble et al., 2020; Mirimin et al., 2019; Rodgers et al., 2018; Seymour & Smith, 2023; Williams et al., 2023). In this work, we adapt a previously published qPCR assay (Mirimin et al., 2019) to assess Arctic charr presence in 15 lochs across Northwestern Scotland. As mentioned, the current Arctic charr records of these lochs are patchy, and traditional monitoring is restricted due to inaccessible terrain and conflict with recreational fishing interests. This study aims to assess the presence of Arctic charr and develop an eDNA monitoring approach for a landscape that will be subject to dramatic anthropogenic change.

## 2 MATERIALS AND METHODS

### 2.1 Study site

**FIGURE 1:**
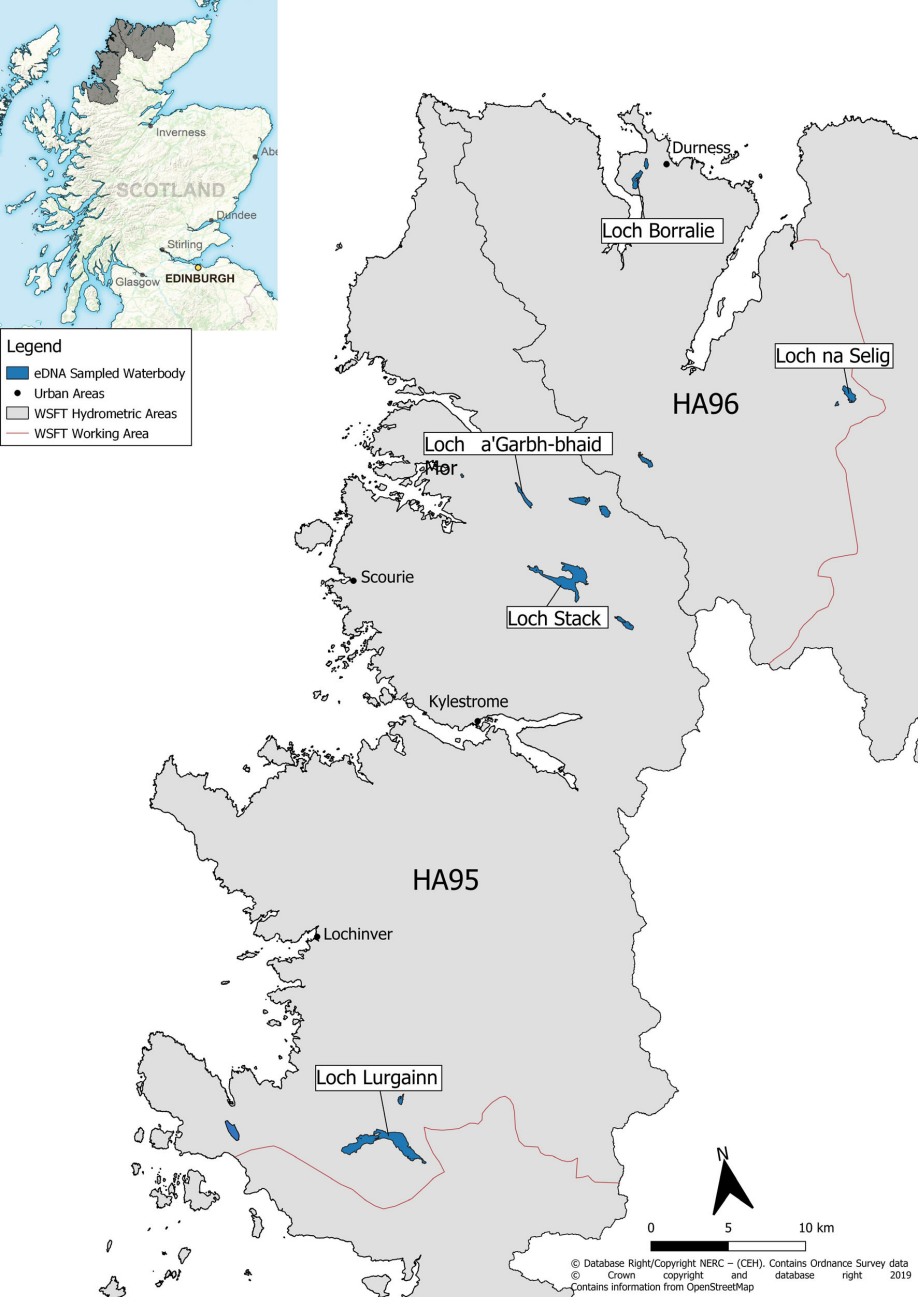
The working area of West Sutherland Fisheries Trust (WSFT) within two hydrometric areas (HA95 and HA96: Laxford and Naver, respectively) in Northwest Scotland. Lochs sampled in the present study are shaded in blue.

We selected 16 sites across 15 study lochs throughout Sutherland in the Northwest Scottish Highlands (Figure 1) based on information presented in Maitland and Adams (2018) and additional sources (S Marshall, personal communication and C Adams, personal communication). Selection criteria included water bodies with anecdotal and/or unconfirmed records for Arctic charr, historic last known records (>10 years since the last survey), localities with a high sensitivity score (Maitland & Adams, 2018), and additional bathymetric data (this paper). The “Maitland and Adams” sensitivity score is a combined measure of altitude, surface area, and bathymetry, where lower altitude and more shallow lochs receive a higher score due to their reduced capacity to physically buffer climate variation (Maitland & Adams, 2018).

We created a “high priority group” of lochs within West Sutherland Fisheries Trust area based on these criteria but excluded lochs exceeding ≈200 hectares (Table S1). We conducted hydroacoustic surveys (Data S1), which provided additional data to assist selection of our priority lochs and inform eDNA sampling locations. Fish communities in these areas naturally exhibit low species richness and include six native freshwater species: Atlantic salmon, brown trout, Arctic charr, European eel (*Anguilla anguilla*), three‐spined stickleback (*Gasterosteus aculeatus*), flounder (*Platichthys flesus*), and one non‐native species, the minnow (*Phoxinus phoxinus*). Special Areas of Conservation designations occur in these areas for freshwater pearl mussels (*Margaritifera margaritifera*) and Atlantic salmon (West Sutherland Fisheries Trust, 2008).

### 2.2 Ethics statement

For three lochs, we compared NORDIC gillnetting with eDNA analysis of Arctic charr presence/absence (see Data S1 for full details of gillnetting). The license for gillnetting was held by Dr. Shona Marshall, West Sutherland Fisheries Trust (Applicant reference CSM‐21‐008, issued by Marine Scotland), and was performed following international netting guidelines (CEN, 2005) for multi‐mesh gillnetting. Care was taken around the timings and depth positions of the nets to avoid the capture of non‐target species such as diving birds. If captured fish individuals were still alive, these were dealt with by being percussion stunned, and then using a knife blade the brain was destroyed, as the net was retrieved.

To test the species specificity of the qPCR assay against closely related species (*S. trutta* and *S. salar*), we non‐lethally harvested fin clips from these species using electrofishing. Individuals selected for non‐lethal fin clips were dosed with MS 222 until Stage 3 anaesthesia was reached (no reaction to handling and loss of buoyancy control). Once Stage 3 anaesthesia was reached, species/fork length was recorded and then no more than 2 mm^2^ of tissue was taken from the dorsal tip of the tail fork using sharp scissors. This was then placed in 100% ethanol vials ready to send on for analysis. Processed fish were then placed into an aerated recovery bucket containing fresh river water and then closely monitored for full recovery. Fish were released back into the covered/habitat rich areas of the sampling reach after 45 min.

### 2.3 Water sampling and eDNA extraction

For a subset of our chosen lochs, sampling stations were selected based on previously collected depth data and focused on the deepest points of the loch or loch outflow (Table S2). In August 2021, we sampled five sites (across four lochs) by collecting water samples at the surface and then at one third and two‐thirds of the total depth of the water body. Loch Lurgainn was sampled at both an east and a west site due to the large size of the loch and the known double‐basin bathymetry. Sampling at these sites involved using a 12‐v field peristaltic pump (series II Geotech, USA) and a 30‐m weighted hose (Masterflex, Cole‐Parmer, USA) to collect up to 2 L of water from depth and to pump directly through a Sterivex capsule filter (Millipore Corp, USA).

In November 2021, we sampled the remaining 11 sites by collecting 2 L of water in sterilized bottles directly from the surface of the outflow stream or immediate shoreline. At a base site, samples were then filtered through a Sterivex capsule filter using a 12‐v field peristaltic pump (series II Geotech). For both sampling trips, at locations where higher turbidity prevented timely filtering of water, filtering was stopped once flow through the filter decreased to less than one drop per second, and the volume of water passed was recorded. Equipment was sterilized between sampling locations by washing in a commercial thin bleach solution (3% sodium hypochlorite) that had been diluted 1:3 parts with water. Filtered volume across the two sampling campaigns ranged from 0.56 to 2 L with a mean of 1.4‐L filtered (Table S1). All water was expelled from the filter units, which were then sealed in individual sterile bags and transported on ice before being frozen at −20°C prior to eDNA extraction. At each sampling location, one field control was also collected, using 1 L of deionized water brought into the field and filtered and stored in the same manner as the eDNA samples.

eDNA sample filters were extracted at Queen Mary University London, UK, using the Qiagen DNeasy Blood & Tissue Kit, following a modified protocol in combination with a Qiagen QIAshredder, based on the methods presented in Doble et al. (2020). Sterile pipe cutters and tweezers were used to remove the filter paper from the Sterivex capsule, which was then torn into strips, and placed into a 1.5‐mL tube. The filter paper was then fully immersed in 450‐μL ATL buffer and 50‐μL Proteinase K, vortexed and incubated for 14–16 h at 56°C and 650 rpm. Following lysis, as much liquid as possible was transferred into a new 1.5‐mL tube, and the remaining filter paper was placed into a QIAshredder spin column and centrifuged for 2 min at 11,000 rpm. The resulting product was added to the previously transferred lysis buffer, 500 μL of AL buffer was added, and the mixture was incubated at 56°C for 10 min. After incubation, 500 μL of 100% ethanol was added, the solution was vortexed, transferred to a DNeasy spin column, and centrifuged for 1 min at 8000 rpm. Transfer to the spin column was repeated until all the lysis solution had been processed. Subsequent steps followed manufacturer's guidelines with a final, double elution in 50‐μL Buffer AE. A negative control, containing no DNA, was included for each batch of extractions and used smaller reagent volumes but otherwise followed the same protocol as the samples. All extracts were stored at −20°C until qPCR analysis.

### 2.4 qPCR Analysis

qPCR analysis was performed at NatureMetrics, Guildford, UK, using the TaqMan assay developed in Mirimin et al. (2019), targeting the mitochondrial cytochrome oxidase subunit 1 (COI) region. The qPCR reaction was conducted in a final reaction volume of 15 μL, comprising 1× TaqMan Environmental Master Mix 2.0 (Applied Biosystems), 0.4 μM forward (5’‐CCCAGCTATTTCTCAATATCAAACC‐3′) and reverse (5’‐GATTTCGGTCCGTGAGTAACA‐3′) primers, 0.1‐μM hydrolysis probe (/56‐FAM/CCCGTTCTAGCAGCAGGCATTACT/3BHQ_1/), 2‐μL DNA sample, 0.2× IPCC Master Mix (Eurogentec), 0.5× IPCC DNA (Eurogentec), and molecular grade water. Cycling conditions consisted of an initial denaturation of 95°C for 10 min followed by 40 cycles of 95°C for 15 s and 60°C for 1 min. qPCR runs were carried out on an CFX Opus384 (BioRad). Each eDNA sample was analysed across 12 replicates. Each plate additionally had triplicate non‐template controls (NTCs) and a triplicate six‐point 1:10 serial dilution of a synthetic DNA positive control (gBlock Gene Fragment, IDT), ranging from 1 × 10^5^ to 1 × 10^0^ copies/μL. This serial dilution was used to generate a standard curve for calculation of assay efficiency and quantification of target DNA in a sample. The Eurogentec Internal Positive Control‐Cy5 (IPCC) was used to test for the presence of PCR inhibitors in our samples. A mean quantification cycle Cq shift of more than two cycles in the environmental sample compared to the controls was considered evidence of inhibition. Amplification in one of the 12 replicates deemed a sample as positive for Arctic charr.

The specificity of the qPCR assay for Scottish Arctic charr was tested using DNA extracted from fin clips of Arctic charr, Atlantic salmon, and brown trout collected at the same time as eDNA sampling using electrofishing surveys and NORDIC gillnet sampling. The limit of detection (LOD) of the assay was also assessed using 20 replicates of a 12‐point 1:2 dilution series of synthetic DNA ranging from 1 × 10^3^ to 0.5 copies/μL. The LOD was considered as the lowest concentration where 95% of the replicates showed positive amplification.

## 3 RESULTS

Environmental DNA successfully detected Arctic charr in 10 of the 16 sites sampled. The assay showed no detection of DNA extracted from salmon and trout fin clips and had an LOD of 3.9 copies/μL. Additionally, the average efficiency of the standard curve was 96.4% (range 86.75–103.59%) with an *R* ^2^ value of 0.998. No amplification occurred in the PCR and field negative controls.

**FIGURE 2:**
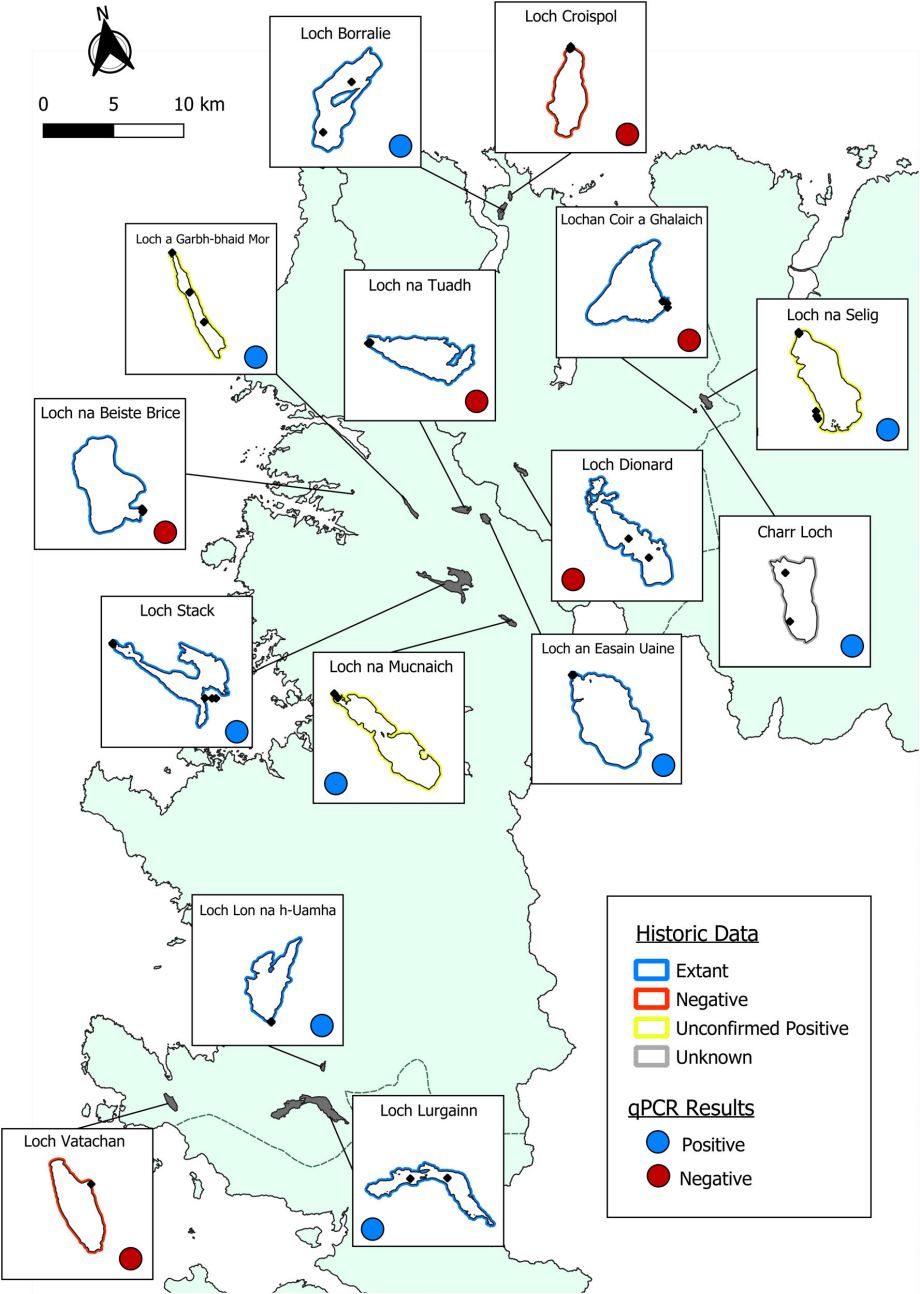
The selected sample sites from across West Sutherland highlighting historic Arctic charr data (Maitland & Adams, 2018) and current eDNA results. Inserts show sampling locations at each site, illustrated with black points, with only Loch Borralie, Loch a'Garbh‐bhaid Mòr, Loch Dionard, and Loch Lurgainn being sampled at depth. Points appearing at a distance from the loch represent sampling at the loch's outflow.

At the two lochs (Loch na Mucnaich and Loch na selig), with previous anecdotal evidence only, we positively amplified Arctic charr eDNA from all three of the field replicates (Figure 2). However, there were also four lochs (Loch Dionard, Loch na Beiste Brice, Loch na Tuadh, and Lochan Coir a'Ghalaich) that were presumed to have extant populations based on latest records, which showed no amplification for target Arctic charr in any of field replicates (Figure 2). Additionally, eDNA results seem to confirm the absence of Arctic charr at the two sites with negative surveys when last recorded (Loch Vatachan and Loch Croispol) (Figure 2).

In six sites, positive amplification was detected in all of the field replicates across multiple depths (Table 1); however, at Loch Borralie, one field replicate at 8 m depth showed no detection, where all other replicates at this site were positive for Arctic charr eDNA. In Loch an Easain Uaine, positive amplification was only seen in the outflow sample and not in either shoreline replicate (Table 1). Additionally, two sites showed inhibition in two of the three samples, with the remainder sample having positive amplification for Arctic charr (Table 1).

**TABLE 1:** Quantitative PCR (qPCR) data for all sampling sites and replicates, including the number of positive amplifications across 12 technical replicates and average Cq values.

Loch name	Number of field replicates	Sampling depth (m)	Sampling position	Number of positive reps (/12)	qPCR Cq (mean ± SD)	Mean ± SD concentration (copies/μL)	Based on Maitland and Adams (2018)	
							Last record	Arctic charr presence
Loch Vatachan	1	1	Outflow	0	No Cq		1995	Uncertain; Neg 1995
	2	1	Outflow	0	No Cq			
	3	1	Outflow	0	No Cq			
Loch Lurgainn East	1	1	Deepest point	9	36.60 ± 0.77	0.45 ± 0.27	1975	Likely extant
	2	1	Deepest point	7	36.38 ± 0.82	0.52 ± 0.26		
	1	15	Deepest point	5	36.93 ± 0.55	0.26 ± 0.09		
	2	15	Deepest point	11	36.44 ± 0.89	1.11 ± 0.68		
	1	30	Deepest point	4	36.95 ± 0.35	0.25 ± 0.07		
	2	30	Deepest point	9	36.59 ± 0.48	0.33 ± 0.12		
Loch Lurgainn West	1	1	Deepest point	3	37.17 ± 0.08	0.26 ± 0.01	1975	Likely extant
	2	15	Deepest point	4	37.03 ± 0.26	0.29 ± 0.06		
	1	15	Deepest point	2	37.52 ± 0.73	0.22 ± 0.11		
	2	30	Deepest point	7	37.63 ± 0.99	0.24 ± 0.21		
	1	30	Deepest point	9	37.23 ± 0.94	0.29 ± 0.2		
Loch Dionard	1	1	Deepest point	0	No Cq		1969	Likely extant
	2	1	Deepest point	0	No Cq			
	1	3	Deepest point	0	No Cq			
	2	3	Deepest point	0	No Cq			
	1	4	Deepest point	0	No Cq			
	2	4	Deepest point	0	No Cq			
	1	6	Deepest point	0	No Cq			
	2	6	Deepest point	0	No Cq			
Loch Borralie	1	1	Deepest point	3	37.49 ± 0.24	0.18 ± 0.03	2009	Extant
	2	1	Deepest point	1	36.84	0.28		
	1	11	Deepest point	2	36.45 ± 0.12	0.37 ± 0.03		
	2	8	Deepest point	0	No Cq			
	1	22	Deepest point	4	36.69 ± 0.43	0.32 ± 0.08		
	2	16	Deepest point	3	36.22 ± 0.98	0.49 ± 0.26		
Loch a’ Garbh‐bhaid Mòr	1	1	Deepest point	6	37.37 ± 0.2	0.23 ± 0.03	1940	Uncertain
	2	1	Deepest point	4	37.25 ± 0.89	0.29 ± 0.17		
	3	1	Outflow	11	36.77 ± 0.91	0.42 ± 0.25		
	1	10	Deepest point	6	37.28 ± 0.69	0.27 ± 0.14		
	2	10	Deepest point	6	37.22 ± 0.6	0.27 ± 0.12		
	1	20	Deepest point	11	36.24 ± 1.1	0.66 ± 0.52		
	2	20	Deepest point	6	37.10 ± 0.73	0.3 ± 0.14		
Loch Croispol	1	1	Outflow	0	No Cq		2009	Uncertain; Neg 2009
	2	1	Shoreline	0	No Cq			
	3	1	Shoreline	0	No Cq			
Loch na Beiste Brice	1	1	Outflow	0	No Cq		1954	Likely extant
	2	1	Shoreline	0	No Cq			
	3	1	Shoreline	0	No Cq			
Loch an Easain Uaine	1	1	Outflow	4	35.82 ± 0.51	0.27 ± 0.11	2002	Extant
	2	1	Shoreline	0	No Cq			
	3	1	Shoreline	0	No Cq			
Loch na Tuadh	1	1	Outflow	0	No Cq		2002	Extant
	2	1	Outflow	0	No Cq			
	3	1	Shoreline	0	No Cq			
Loch na Mucnaich	1	1	Outflow	10	36.54 ± 0.91	1.3 ± 0.84	Anecdotal	Unconfirmed
	2	1	Outflow	3	37.31 ± 1.21	0.82 ± 0.63		
	3	1	Shoreline	11	35.98 ± 0.93	1.87 ± 1.19		
Loch Lon na h‐Uamha	1	1	Outflow	11	36.49 ± 0.84	1.28 ± 0.6	1963	Likely extant
	2	1	Outflow	12	36.69 ± 0.7	1.08 ± 0.4		
	3	1	Shoreline	12	35.61 ± 0.54	2.12 ± 0.65		
Loch Stack	1	1	Outflow	9	37.41 ± 0.62	0.53 ± 0.21	2002	Extant
	2	1	Outflow	Inhibited				
	3	1	Shoreline	Inhibited				
Lochan Coir a'Ghalaich	1	1	Outflow	0	No Cq		2001	Extant
	2	1	Shoreline	0	No Cq			
	3	1	Shoreline	0	No Cq			
Charr loch	1	1	Shoreline	11	35.13 ± 1.08	2.81 ± 1.75	‐	‐
	2	1	Shoreline	Inhibited				
	3	1	Shoreline	Inhibited				
Loch na Seilg	1	1	Outflow	12	34.33 ± 0.61	1.60 ± 0.54	Anecdotal	Unconfirmed
	2	1	Outflow	10	35.95 ± 0.68	0.54 ± 0.27		
	3	1	Shoreline	12	34.54 ± 0.77	1.45 ± 0.68		

*Note*: Historical records based on Maitland and Adams (2018) are also presented. The status “unconfirmed” for Arctic charr presence indicates that no scientific investigation has taken place.

Across all of the sites with positive eDNA detection, the mean concentration of DNA was low, ranging between 0.18 and 2.81 copies/μL (Table 1). These values were lower than the calculated LOD for the assay, despite having mean Cq values less than 38, and were often below the minimum standard curve concentration of 1 copy/μL, meaning that eDNA concentrations were extrapolated.

## 4 DISCUSSION

This study aimed to assess the use of a targeted qPCR approach for eDNA monitoring of Arctic charr in lochs across Northwest Scotland, a landscape with little data regarding the true extent of Arctic charr presence yet vulnerable to rapid warming in the upcoming years. The qPCR approach successfully detected positive DNA from 10 sites. These ranged from sites previously identified with anecdotal evidence to known populations with historic records of varying ages across Northwest Scotland. This approach also confirmed negative historic survey results from two lochs. The work highlights the success of both outflow‐shore based sampling and water column sampling to detect Arctic charr eDNA, with both methods showing positive detections in lochs with known extant populations of Arctic charr.

The successful shoreline sampling aligns well with the existing body of knowledge regarding cold‐water salmonid monitoring with eDNA. Previous work by Lawson‐Handley et al. (2019) suggests that to enable Arctic charr detection, shoreline samples should be collected at regular intervals due to Arctic charr's nature as a primarily hypolimnion dwelling fish. This process can be costly and time‐consuming due to the volume of samples required to assess a single water body. A recent study by Seymour and Smith (2023) also successfully detected Arctic charr eDNA using shore‐based sampling of known Welsh populations. They concluded that variation in detection strength was influenced by population ecology rather than spatial location, although they agree with Lawson‐Handley et al. (2019) regarding the importance of broadening the spatial sampling for Arctic charr eDNA. This could be mitigated by the use of outflow‐shore based sampling, which allows lochs to be spot surveyed but still enables Arctic charr eDNA detection, as shown in this study. Additionally, it enables sampling of remote and boatless lochs that have been previously inaccessible (as are many freshwater habitats of conservation importance).

The current work compliments the findings of Sellers et al. (2024), which found that a mean of 5.37 winter shoreline samples were sufficient to reach the minimum number of samples required to sample 95% of complete species richness across their survey of 101 British lakes. Equal occupancy between shoreline and offshore samples for *Salvelinus* spp. was also highlighted, regardless of the surface area of the water body. Arctic charr's known distribution at depth, outside of spawning periods, could result in reduced eDNA concentration at the surface and thus increase the risk of false‐negative detections. The optimal outflow or shoreline sampling time should thus align with regional Arctic charr spawning (September–January, Walker (2006)), which was the case for some lakes in this study, to increase the likelihood of detection. This timing also coincides with water mixing during the breakdown of the thermocline, which in summer months can keep some eDNA trapped in the deep, hypolimnion layer (Littlefair et al., 2021). Despite the success in this study, it is important to consider how false positives and negatives might be influenced by eDNA degradation, eDNA transport within a catchment, and factors influencing the movement of eDNA downstream to outflow sites. DNA degradation in freshwater environments is complex (Strickler et al., 2015) but could result in a lack of detection at outflow sites despite the presence of the species in the loch. Factors such as salinity, pH, temperature, and microbial activity can all influence the rate of degradation (Saito & Doi, 2021) although we did not measure them in this study. Jacobsen et al. (2023) found that in smaller lakes the levels of Arctic charr eDNA were greater in downstream riverine sampling sites, whereas for larger lakes the quantity of eDNA in downstream sites was reduced compared to upstream sites, potentially due to eDNA retention in the lake. It is worth considering all of these factors when interpreting the negative Arctic charr detections at sites with presumed extant populations such as Lochan Coir a'Ghalaich, Loch na Tuadh, and Loch na Beiste Brice.

Using a paired sampling approach not only enables eDNA ground‐truthing but also provides an insight into the population dynamics of captured individuals, which can only be obtained through traditional destructive survey methods (Di Muri et al., 2022). Only three lochs had NORDIC gillnet investigations conducted during the study (Table S3a,b), resulting in the capture of both trout and Arctic charr. The only loch where all three techniques (outflow‐shore‐based sampling, water column sampling, and NORDIC gillnetting) were used was Loch a’ Garb‐bhaid Mòr. Previous records from this site are the oldest in the study, dating back to 1940, but the results presented in this manuscript allow this record to be updated to 2021. Sixteen Arctic charr individuals were captured through the netting effort, and significant positive eDNA presence was detected through both water column and shoreline sampling. This highlights the potential of the method shown in the present study. Interestingly, Loch Tuadh returned no positive Arctic charr captures when subjected to NORDIC gillnetting and negative eDNA results despite being immediately downstream of Loch an Easain Uaine. Loch an Easain Uaine returned a positive eDNA outflow sample, and through field observations, three salmonid reds were seen surrounding the outflow in the littoral gravels. Based on Maitland and Adams (2018), these two lochs would be candidates for future investigation using these methods.

Given the previously unconfirmed/anecdotal nature of the Arctic charr records, it was heartening to positively detect Arctic charr presence in both Loch na Mucniach and na Seilg for the first time using eDNA. This is particularly significant in Loch na Seilg, given its size and high altitude, which may provide some mitigation against climate change (although its bathymetry remains unknown). Another long‐term record update was achieved for Loch Lon na h‐Uamha, from 1963 to 2021, with significant concentration of eDNA detected from both shoreline and outflow locations. Additionally, Loch Stack's record was updated from 2002 to 2021. This is one of the few sites where Arctic charr are regularly caught and reported by recreational anglers while targeting brown trout. Although sample inhibition occurred, Arctic charr presence was further confirmed by video footage (Figure S2b) of spawning within a Lock Stack tributary burn captured by Chris Conroy (Atlantic Salmon Trust).

Although this study demonstrates the potential of eDNA for monitoring Arctic charr in this region, future work on the UK's Arctic charr populations should focus on lochs with more recent records, lochs downstream of sites with known records, and occasional spawning streams used by known populations. It would also be prudent to establish water column monitoring in the area to provide crucial data on the duration and periods of stratification in lochs, and to address the current data deficiency in thermocline knowledge and its effects on isolated Arctic charr populations, both currently and in the future.

More broadly, emphasis should be given on expanding population maps not only for Arctic charr but also for other species vulnerable to climate change. This is of particular importance based on simulated climate change scenarios such as those presented in Kelly et al. (2020), which project increases of 3–4°C in European lakes toward century end. Additionally, our findings will be relevant to more northerly sites as warming continues to affect boreal regions. Cold‐water fish species could decline if such increases are seen, as they are more likely to be exposed to temperatures above their threshold tolerance. Furthermore, warmer waters may bring new species to the habitat and force species to leave their natural ranges (Bommersbach et al., 2024). Arctic charr may thus be exposed to new competitors (Falardeau et al., 2022) or be forced to move to colder, deeper habitats, which might not be possible in isolated lochs. Consequently, it is vital that monitoring strategies are in place to track Arctic charr populations throughout their range to enable mitigation against water warming where possible.

Overall, environmental DNA is rapidly becoming an essential tool for effective management programmes and can be used in conjunction with traditional methods to ensure comprehensive monitoring across a catchment. This is particularly important given the need for efficient monitoring efforts, which have been traditionally hindered by requirements for access approvals and restrict timely sampling. The eDNA methodology presented here, using shore and outflow sampling, overcomes these limitations and offers an Arctic charr monitoring method better suited for rapid response to climate change, vital for a species that is highly vulnerable to rising lake temperatures.

## AUTHOR CONTRIBUTIONS

Jane Hallam and Samuel J Poultney designed the project and carried out fieldwork for eDNA. Samuel J Poultney carried out NORDIC and Bathymetry fieldwork. Jane Hallam and Tianna E. Hewitson extracted eDNA samples. Molly Ann Williams and Tianna E. Hewitson conducted qPCR laboratory analysis. Molly Ann Williams analysed qPCR data. Joanne E. Littlefair supervised the project and sourced funding. Molly Ann Williams and Samuel J Poultney devised the first draft of the manuscript. All authors have contributed to the final version of the manuscript.

## FUNDING INFORMATION

This work was funded by the West Sutherland Fisheries Trust, Queen Mary University of London, and The Fishmongers’ Company targeted academic sponsorship. NatureMetrics purchased reagents for the project.

## Supporting information


**DATA S1.** Supporting information.

## Data Availability

The data that support the findings of this study are available in the manuscript and supplementary material of this article.
