# Supplementary material for: From anecdotes to evidence: Environmental DNA detection of Arctic charr (Salvelinus alpinus L.) at the southern limit of its circumpolar range
Source: J Fish Biol. 2025 Jan 22;107(1):71–81. doi: 10.1111/jfb.16048 (PMC12327159; doi:10.1111/jfb.16048)
Supplement: Supplementary file 1 — DATA S1. Supporting information. [file JFB-107-71-s001.docx]

From anecdotes to evidence: Environmental DNA detection of Arctic charr (*Salvelinus alpinus* L.) at the southern limit of its circumpolar range

# Author list

Molly Ann Williams^*^, Samuel J Poultney^*^, Jane Hallam, Tianna E Hewitson, Joanne E Littlefair^†^

^*^ Joint first author

^†^ Corresponding author

# S1 Supplementary Methods

# S1.1 Bathymetric and NORDIC Gill Net Investigations

S1.1.1 Bathymetric Investigation

Prior to the main investigation, a preliminary bathymetric survey took place across all the selected water bodies to locate the deepest areas. For isolated or boat-less locations, an inflatable raft was used as a platform to perform the depth soundings using a handheld depth sounder (Speedtech Instruments, VA USA) and a consumer GPS (eTrex 20x; [www.garmin.com](http://www.garmin.com)). Due to the lack of controlablity of the raft; transects were dictated by wind direction on a given day, creating drifting linear transects amounting to one longitudinal transect and between one to six lateral transects. A total of thirteen lochs were surveyed using this method. For locations which had boat access available, a hydroacoustic system was used to generate an accurate GPS fixed bathymetry and additional software information (i.e. bottom hardness, macrophyte cover and height). The hydroacoustic system used was a Lowrance Hook Reveal 5 echo sounder operating on ‘automatic’ at between 50-200 kHz (Lowrance; [www.lowrance.com](http://www.lowrance.com/)); with the transducer deployed to the depth of the keel and at a speed of ≤4 km/h. See Miller *et al*., (2015) and Winfield *et al*., (2015) for a full system summary and its ground-truthing application.

To generate an accurate coverage, the sounder was passed in an approximate continuous diagonal track pattern (Bean, 2003). For water bodies with existing bathymetry data (i.e. Murray & Pullar, 1910, available here: <https://maps.nls.uk/view/74422159>), the continuous diagonal track pattern was also used across the deepest area/location. The data was recorded onto a Kingston 32GB, 60MB/s micro SDHC card which was uploaded to the BioBase system immediately after fieldwork. Boat access limited the deployment of the hydroacoustic system to seven lochs

## S1.1.2 NORDIC Gill Netting

Gill net sampling was informed by individual stakeholder consultation on access and gillnet fishing permissions. Appelberg *et al*., (1995) and Bean, (2003) were used to inform NORDIC gill netting suitability and the minimum number of nets to deploy. Depending on the suitability of the survey area, one to four NORDIC gill nets were deployed in a stratified sampling design in accordance to CEN, (2005). The 1-3m strata was omitted from the survey design to avoid capture of non-target species such as diving birds. One net was set at each net layer which focused around 3-6; 6-12 and 12-20m with only one net set at 20-35m based on the corresponding depth survey (i.e Loch a’Garbh-bhaid Mòr). The nets were deployed perpendicular to the shoreline for a 12hr period. A GPS position of the approximate start and end points of the net were noted, along with depth and secchi depth. Deployment and retrieval time was also recorded.

## S1.2 Data Analysis

### S1.2.1 Bathymetry

The data analysis was performed by the BioBase hydroacoustic analysis system (BioBase V5.2; [www.biobasemaps.com](http://www.biobasemaps.com)). Using BioBase, the hydroacoustic data and GPS positions from the echo sounder were analysed to generate standardised software outputs : 1) depth, 2) macrophyte presence/absences, 3) macrophyte height and 4) sediment/bottom hardness. Only depth data was extracted from BioBase as a shapefile and imported into a project specific GIS layer (version 3.16.3 Hannover; [www.qgis.org](http://www.qgis.org/)) to enable data presentation and netting positions.

## S2. Supplementary Results

### S2.1 Bathymetry

By using a manual, handheld sounder; data points ranged from 29 (Loch an Mucnaich) to 253 (Loch Vatachan) due to the size of the waterbody surveys and the amount of drifting linear transects conducted. Due to the more accurate Lowrance sounder, data points were much greater (≥4916), with each survey producing a percentage coverage between 38.1% (Loch Bad a’Ghaill) and 100% (Loch na Dail) depending on the survey area/waterbody. A summary of data captured during the fieldwork using both methods is shown in Table S2.

Given the requirement for a stable, controllable survey platform during echo sounding, lochs were surveyed in accordance with the forecasted/observed wind speed with an upper limit of 4m/s. Data corruption was encountered whilst performing echo sounding transects on Loch a'Garbh-bhaid Beag but the survey was merely repeated at a later date. Six lochs were not surveyed either due to access permissions, mechanical or equipment issues or time constraints. These are Loch an Leathaid Bhuain, Loch Croispol, Loch na Seilg, "The Charr Loch" and Lochan Coir a’Ghalaich.

Outside of Murray & Pullar, (1910) survey data and anecdotal depth information from anglers, the accurate, high resolution bathymetric data collected during this piece of work is new knowledge especially for isolated and/or high altitude lochans in West Sutherland. Data collected via the Lowrance equipment is freely available in an interactive map form via C-Map Genesis Social Map (<https://www.genesismaps.com/socialmap/>).

### S2.2 NORDIC Gill Netting

Table S3 gives an overview of the results from the netting sessions across August 2021. Sechi depths are as follows for Mathair a'Gharbh Uilt, Loch a'Garbh-bhaid Mor and Loch na Tuadh respectively, 2.5m, 3.5m and 3.6m. Various degrees of overnight predation occurred on fish captured within the nets across all survey sites, potentially due to the eel population present in these waterbodies.

Mathair a’Gharbh Uilt captured twelve brown trout individuals across two net depths on the night of the 7^th^ August. Individuals ranged from 142mm to 222mm in length and 33-117g in weight. It is interesting to note that a large mature eel was tangled and subsequently released from Net #2.

The netting of Loch a'Garbh-bhaid Mòr (Night of 19^th^ August) produced twelve brown trout and sixteen charr specimens between two net depths of 6-7m and 12-16m respectively. No individuals were captured at the third net depth between 25-32m. Length and weight of brown trout captured ranged between 114-240mm and 20-165g compared with charr between 95-176mm and 11-64g respectively. A full breakdown of charr individuals can be seen in Table S4. For additional investigations into UK charr populations, morphometric photographs and tissue samples were also collected/recorded and sent on to the relevant laboratories The largest specimen captured was a 5-0 yr male, brown trout at 240mm and 165g captured from Loch a’Garbh-bhaid Mòr.

Loch na Tuadh (Night of 21^st^ August), produced nineteen brown trout individuals from between 95-234mm and 11-133g from only one net depth (3-5m). Whilst retrieving the nets, two specimens were lost.

**S3. Supplementary Tables and Figures**

**Table S1:** Master environmental DNA sampling data sheet.

| Date | Loch | Lat | Long | Air temp (°C) | Water temp (°C) | Depth (m) | SA/ha | Altitude | Sample | Volume (ml) | Notes |
| --- | --- | --- | --- | --- | --- | --- | --- | --- | --- | --- | --- |
| 13/08/2021 | Loch Vatachan | 58.04645 | -5.356184 | 15 | 14.4 | 1 | 54 | 8 | 1 | 1300 | Three surface samples from the outlet leading to Loch Ra |
| 13/08/2021 | Loch Vatachan | 58.04645 | -5.356184 | 15 | 14.4 | 1 | 54 | 8 | 2 | 1300 |  |
| 13/08/2021 | Loch Vatachan | 58.04645 | -5.356184 | 15 | 14.4 | 1 | 54 | 8 | 3 | 1300 |  |
| 13/08/2021 | **Blank control** | | | | | | | | | 1000 |  |
| 14/08/2021 | Loch Lurgainn EAST | 58.02828 | -5.18645 | 14 | 15.3 | 1 | 326 | 56 | 1 | 1900 | Clear |
| 14/08/2021 | Loch Lurgainn EAST | 58.02828 | -5.18645 | 14 | 15.3 | 1 | 326 | 56 | 2 | 1850 |  |
| 14/08/2021 | Loch Lurgainn EAST | 58.02828 | -5.18645 | 14 | 15.3 | 15 | 326 | 56 | 1 | 1900 |  |
| 14/08/2021 | Loch Lurgainn EAST | 58.02828 | -5.18645 | 14 | 15.3 | 15 | 326 | 56 | 2 | 1900 |  |
| 14/08/2021 | Loch Lurgainn EAST | 58.02828 | -5.18645 | 14 | 15.3 | 30 | 326 | 56 | 1 | 1850 |  |
| 14/08/2021 | Loch Lurgainn EAST | 58.02828 | -5.18645 | 14 | 15.3 | 30 | 326 | 56 | 2 | 2000 |  |
| 14/08/2021 | Loch Lurgainn WEST | 58.02801 | -5.21286 | 14 | 16 | 1 | 326 | 56 | 1 | 2000 | Bad weather coming in |
| 14/08/2021 | Loch Lurgainn WEST | 58.02801 | -5.21286 | 14 | 16 | 15 | 326 | 56 | 2 | 2000 |  |
| 14/08/2021 | Loch Lurgainn WEST | 58.02801 | -5.21286 | 14 | 16 | 15 | 326 | 56 | 1 | 1800 |  |
| 14/08/2021 | Loch Lurgainn WEST | 58.02801 | -5.21286 | 14 | 16 | 30 | 326 | 56 | 2 | 1850 |  |
| 14/08/2021 | Loch Lurgainn WEST | 58.02801 | -5.21286 | 14 | 16 | 30 | 326 | 56 | 1 | 2000 |  |
| 14/08/2021 | **Blank control** | | | | | | | | | 1000 |  |
| 15/08/2021 | Loch Dionard | 58.39736 | -4.81044 | 13 | 14.5 | 1 | 25 | 107 | 1 | 950 | Raining on and off |
| 15/08/2021 | Loch Dionard | 58.39736 | -4.81044 | 13 | 14.5 | 1 | 25 | 107 | 2 | 900 |  |
| 15/08/2021 | Loch Dionard | 58.39736 | -4.81044 | 13 | 14.5 | 3 | 25 | 107 | 1 | 900 |  |
| 15/08/2021 | Loch Dionard | 58.39736 | -4.81044 | 13 | 14.5 | 3 | 25 | 107 | 2 | 900 |  |
| 15/08/2021 | Loch Dionard | 58.39736 | -4.81044 | 13 | 14.5 | 6 | 25 | 107 | 1 | 950 |  |
| 15/08/2021 | Loch Dionard | 58.39736 | -4.81044 | 13 | 14.5 | 6 | 25 | 107 | 2 | 1000 |  |
| 15/08/2021 | Loch Dionard | 58.39905 | -4.81388 | 13 | 14.5 | 4 | 25 | 107 | 1 | 850 |  |
| 15/08/2021 | Loch Dionard | 58.39905 | -4.81388 | 13 | 14.5 | 4 | 25 | 107 | 2 | 900 |  |
| 15/08/2021 | **Blank control** | | | | | | | | | 1000 |  |
| 29/09/2021 | Loch Borralie | 58.55832 | -4.785703 | 10 | 13.5 | 1 | 35 | 17 | 2 | 2000 | Waypoint (1654) |
| 29/09/2021 | Loch Borralie | 58.55832 | -4.785703 | 10 | 13.5 | 8 | 35 | 17 | 2 | 2000 |  |
| 29/09/2021 | Loch Borralie | 58.55832 | -4.785703 | 10 | 13.5 | 16 | 35 | 17 | 2 | 2000 |  |
| 29/09/2021 | Loch Borralie | 58.56348 | -4.780128 | 10 | 13.5 | 1 | 35 | 17 | 1 | 2000 | Waypoint (1655) |
| 29/09/2021 | Loch Borralie | 58.56348 | -4.780128 | 10 | 13.6 | 11 | 35 | 17 | 1 | 2000 |  |
| 29/09/2021 | Loch Borralie | 58.56348 | -4.780128 | 10 | 13.6 | 22 | 35 | 17 | 1 | 2000 |  |
| 29/09/2021 | **Blank control** | | | | | | | | | 1000 | Sunshine and occasional heavy showers |
| 06/10/2021 | Loch a' Garbh-bhaid Mòr | 58.38587 | -4.950416 | 14 | 15 | 1 | 34 | 56 | 2 | 1550 |  |
| 06/10/2021 | Loch a' Garbh-bhaid Mòr | 58.38587 | -4.950416 | 14 | 15 | 10 | 34 | 56 | 2 | 1350 |  |
| 06/10/2021 | Loch a' Garbh-bhaid Mòr | 58.38587 | -4.950416 | 14 | 15 | 20 | 34 | 56 | 2 | 1200 |  |
| 06/10/2021 | Loch a' Garbh-bhaid Mòr | 58.39034 | -4.954589 | 14 | 15 | 1 | 34 | 56 | 1 | 1000 |  |
| 06/10/2021 | Loch a' Garbh-bhaid Mòr | 58.39034 | -4.954589 | 14 | 15 | 10 | 34 | 56 | 1 | 1000 |  |
| 06/10/2021 | Loch a' Garbh-bhaid Mòr | 58.39034 | -4.954589 | 14 | 15 | 20 | 34 | 56 | 1 | 900 |  |
| 06/10/2021 | Loch a' Garbh-bhaid Mòr | 58.06331 | -5.007041 | 14 | 4 | 1 | 34 | 56 | Outflow | 1000 | Exsitu Filtering @19:55 |
| 06/10/2021 | **Blank control** | | | | | | | | | 1000 |  |
| 23/11/2021 | Loch Croispol | 58.57337 | -4.769009 | 9 | 4 | 1 | 10 | 14 | Outflow | 2000 | (1656) Exsitu Filtering @ 13:49 Stored in fridge prior to filtering |
| 23/11/2021 | Loch Croispol | 58.57324 | -4.768912 | 9 | 4 | 1 | 10 | 14 | Shoreline | 2000 | (1657) Exsitu Filtering @ 14:15 |
| 23/11/2021 | Loch Croispol | 58.57319 | -4.769149 | 9 | 4 | 1 | 10 | 14 | Shoreline | 2000 | (1658) Exsitu Filtering @ 14:48 |
| 23/11/2021 | **Blank control** | | | | | | | | | 1000 | Sun and Shower Intervals: Westerly, 8m/s All bottles rinsed 3 times at sampling locations. |
| 23/11/2021 | Loch na Beiste Brice | 58.4037 | -5.015425 | 9 | 4 | 1 | 3 | 47 | Outflow | 1150 | (1659) Exsitu Filtering @ 15:45 |
| 23/11/2021 | Loch na Beiste Brice | 58.40366 | -5.015456 | 9 | 4 | 1 | 3 | 47 | Shoreline | 1100 | (1660) Exsitu Filtering @ 16:05 |
| 23/11/2021 | Loch na Beiste Brice | 58.40374 | -5.01548 | 9 | 4 | 1 | 3 | 47 | Shoreline | 1000 | (1661) Exsitu Filtering @16:24 |
| 23/11/2021 | **Blank control** | | | | | | | | | 1000 | Sun and Shower Intervals: SWesterly, 6m/s |
| 25/11/2021 | Loch an Easain Uaine | 58.37622 | -4.870918 | 3 | 4 | 1 | 34 | 196 | Outflow | 2000 | (1668) Exsitu Filtering @ 19:27 |
| 25/11/2021 | Loch an Easain Uaine | 58.37622 | -4.870678 | 3 | 4 | 1 | 34 | 196 | Shoreline | 2000 | (1669) Exsitu Filtering @ 19:45 |
| 25/11/2021 | Loch an Easain Uaine | 58.37621 | -4.870849 | 3 | 4 | 1 | 34 | 196 | Shoreline | 2000 | (1670) Exsitu Filtering @20:04 |
| 25/11/2021 | **Blank control** | | | | | | | | | 1000 | Frequent light rain and hail showers: W, 3m/s |
| 25/11/2021 | Loch na Tuadh | 58.38254 | -4.903341 | 3 | 4 | 1 | 42 | 167 | Outflow | 1700 | (1671) Exsitu Filtering @ 20:38 |
| 25/11/2021 | Loch na Tuadh | 58.38249 | -4.903047 | 3 | 4 | 1 | 42 | 167 | Outflow | 1700 | (1672) Exsitu Filtering @ 21:17 |
| 25/11/2021 | Loch na Tuadh | 58.38263 | -4.90287 | 3 | 4 | 1 | 42 | 167 | Shoreline | 1650 | (1673) Exsitu Filtering @ 21:43 |
| 25/11/2021 | **Blank control** | | | | | | | | | 1000 | Frequent light rain and hail showers: W, 3m/s |
| 26/11/2021 | Loch na Mucnaich | 58.31148 | -4.870089 | 1 | 4 | 1 | 10 | 157 | Outflow | 850 | (1674) Exsitu Filtering @ 18:37 |
| 26/11/2021 | Loch na Mucnaich | 58.31143 | -4.870033 | 1 | 4 | 1 | 10 | 157 | Outflow | 850 | (1675) Exsitu Filtering @ 19:00 |
| 26/11/2021 | Loch na Mucnaich | 58.31108 | -4.869475 | 1 | 4 | 1 | 10 | 157 | Shoreline | 850 | (1676) Exsitu Filtering @ 19:23 |
| 26/11/2021 | **Blank control** | | | | | | | | | 1000 | Sleet Showers: NW, 6m/s |
| 29/11/2021 | Loch Lon na h-Uamha | 58.04602 | -5.172238 | 6 | 4 | 1 | 14 | 87 | Outflow | 650 | (1692) Exsitu Filtering @ 19:14 |
| 29/11/2021 | Loch Lon na h-Uamha | 58.04608 | -5.172227 | 6 | 4 | 1 | 14 | 87 | Outflow | 650 | (1693) Exsitu Filtering @ 19:33 |
| 29/11/2021 | Loch Lon na h-Uamha | 58.04605 | -5.172326 | 6 | 4 | 1 | 14 | 87 | Shoreline | 1000 | (1694) Exsitu Filtering @ 19:51 |
| 29/11/2021 | **Blank control** | | | | | | | | | 1000 | Heavy Rain: SW, 9m/s |
| 29/11/2021 | Loch Stack | 58.34697 | -4.958587 | 9 | 4 | 1 | 257 | 36 | Outflow | 1100 | (1695) Exsitu Filtering @ 21:09 |
| 29/11/2021 | Loch Stack | 58.34669 | -4.958376 | 9 | 4 | 1 | 257 | 36 | Outflow | 1100 | (1696) Exsitu Filtering @ 21:23 |
| 29/11/2021 | Loch Stack | 58.34707 | -4.958356 | 9 | 4 | 1 | 257 | 36 | Shoreline | 1100 | (1697) Exsitu Filtering @ 21:40 |
| 29/11/2021 | **Blank control** | | | | | | | | | 1000 | Rain Showers: SW, 6m/s |
| 28/11/2021 | Lochan Coir a'Ghalaich | 58.41646 | -4.59327 | -2 | 4 | 1 | 4 | 536 | Outflow | 2000 | (1680) Exsitu Filtering @ 18:17 |
| 28/11/2021 | Lochan Coir a'Ghalaich | 58.41652 | -4.593531 | -2 | 4 | 1 | 4 | 536 | Shoreline | 2000 | (1681) Exsitu Filtering @ 18:38 |
| 28/11/2021 | Lochan Coir a'Ghalaich | 58.41634 | -4.593244 | -2 | 4 | 1 | 4 | 536 | Shoreline | 2000 | (1682) Exsitu Filtering @ 18:52 |
| 28/11/2021 | **Blank control** | | | | | | | | | 1000 | Sunny- Occasional Snow Showers: N, 3m/s *Empheral Outflow |
| 28/11/2021 | Charr Loch | 58.41784 | -4.582 | -2 | 4 | 1 | NR | NR | Shoreline | 2000 | (1683) Exsitu Filtering @ 19:14 |
| 28/11/2021 | Charr Loch | 58.41813 | -4.582296 | -2 | 4 | 1 | NR | NR | Shoreline | 2000 | (1684) Exsitu Filtering @ 19:30 |
| 28/11/2021 | Charr Loch | 58.41862 | -4.582382 | -2 | 4 | 1 | NR | NR | Shoreline | 2000 | (1685) Exsitu Filtering @ 19:43 |
| 28/11/2021 | **Blank control** | | | | | | | | | 1000 | Sunny- Occasional Snow Showers: N, 3m/s *No Outflow |
| 28/11/2021 | Loch na Seilg | 58.42667 | -4.585765 | -1 | 4 | 1 | 40 | 396 | Outflow | 1750 | (1686) Exsitu Filtering @ 20:14 |
| 28/11/2021 | Loch na Seilg | 58.42656 | -4.585637 | -1 | 4 | 1 | 40 | 396 | Outflow | 1750 | (1687) Exsitu Filtering @ 20:35 |
| 28/11/2021 | Loch na Seilg | 58.42649 | -4.5857 | -1 | 4 | 1 | 40 | 396 | Shoreline | 1750 | (1688) Exsitu Filtering @ 20:57 |
| 28/11/2021 | **Blank control** | | | | | | | | | 1000 | Sunny- Occasional Snow Showers: N, 3m/s |

**Table S2**: The priority waterbodies bathymetry investigation results alongside sampling methods for Arctic charr eDNA. Given the different in degrees of accuracy and complete coverage between the two methods, depth is a taken as observed depth rather than absolute depth

| **Waterbody** | **Survey Date** | **Data Points** | **Mean Depth/m** | **Min. Depth/m** | **Max. Depth/m** | **Gillnet Sampled** | **eDNA Sampled** |
| --- | --- | --- | --- | --- | --- | --- | --- |
| Loch Vatachan * | 05/06/2021 | 253 | 8.74 | ≤0.4 | 15.5 |  | YES |
| Loch Bad a'Ghaill † | 11/07/2021 | 141710 | 15.45 | 0.9 | 58.7 |  |  |
| Loch Bad na h-Achlaise * | 11/07/2021 | 213 | 11.00 | 0.7 | 18.0 |  |  |
| Loch Lurgainn † | 03/07/2021 | 178689 | 22.05 | 1.5 | 50.0 |  | YES |
| Loch Uidh Tarraigean * | 09/07/2021 | 199 | 16.69 | 0.7 | 35.8 |  | YES |
| Loch na Dail † | 09/07/2021 | 4976 | 5.76 | 1.0 | 15.8 |  | YES |
| Loch Lon na h-Uamha ***** | 21/07/2021 | 176 | 8.02 | ≤0.4 | 25.5 |  | YES |
| Loch Urigill | 1902 | 124 | 3.04 | 0.3 | 12.1 |  |  |
| Loch a'Bharaille * | -- | | | | |  |  |
| Loch an Leathaid Bhuain † | -- | | | | |  | YES |
| Lochain Doimhain * | 29/05/2021 | 105 | 3.86 | ≤0.4 | 9.3 |  |  |
| Loch Eileanach * | 29/05/2021 | 76 | 1.68 | ≤0.4 | 4.5 |  |  |
| Loch Stack | 1902 | 183 | 11 | 0.3 | 33 |  | YES |
| Loch an Smuraich * | 28/05/2021 | 34 | 3.57 | ≤0.4 | 9.8 |  | YES |
| Loch na Mucnaich * | 22/05/2021 | 29 | 8.40 | 0.9 | 9.6 |  | YES |
| Loch a’Garbh-bhaid Mòr † | 29/06/2021 | 42051 | 9.33 | 0.5 | 31.2 | YES | YES |

| **Waterbody** | **Survey Date** | **Data Points** | **Mean Depth/m** | **Min. Depth/m** | **Max. Depth/m** | **Gillnet Sampled** | **eDNA Sampled** |
| --- | --- | --- | --- | --- | --- | --- | --- |
| Loch a'Garbh-bhaid Beag † | 02/07/2021 | 13010 | 1.01 | 0.5 | 6.5 |  |  |
| Mathair a'Gharb Uilt * | 31/05/2021 | 183 | 3.45 | 0.6 | 9.3 | YES | YES |
| Loch na Beiste Brice * | 28/05/2021 | 36 | 2.99 | ≤0.4 | 5.4 |  | YES |
| Loch na Tuadh † | 15/06/2021 | 4916 | 6.72 | 0.6 | 17.2 | YES | YES |
| Loch an Easain Uaine* | 20/06/2021 | 190 | 8.37 | ≤0.4 | 19.0 |  | YES |
| Loch Dionard † | 27/06/2021 | 35623 | 2.99 | 0.6 | 8.3 |  | YES |
| Coire Duail Lochan * | 06/06/2021 | 65 | 1.55 | ≤0.4 | 4.1 |  |  |
| Loch Borralie † | 12/09/2021 | 52685 | 10.74 | 0.5 | 35.6 |  | YES |
| Loch Croispol † | -- | | | | |  | YES |
| Loch na Seilg * | -- | | | | |  | YES |
| “The Charr Loch”* | -- | | | | |  | YES |
| Loch a'Ghobha-Dhuibh * | -- | | | | |  |  |
| Lochan Coir a’Ghalaich * | -- | | | | |  | YES |
| * Denotes handheld sounder; † denotes Lowrance sounder, -- denotes missing data. Murray & Pullar, (1910) data is denoted in *italics*. | | | | | |  |  |

**Table S3.** NORDIC Multi Mesh gill net results. [Date of Survey Night: 07/08/21, 19/08/21 and 21/08/21 respectively]. Additional locations were selected for investigation but were not completed; namely Loch an Easain Uaine, Loch an Leathaid Bhuain and Loch na Mucnaich. Maturity classification is based on Kesteven, (1960). Age Determination based on scale reading and otolith analysis for trout and charr respectively.

| **Waterbody** | **Net #** | | **Net Depth**  **(m)** | **Species** | **No. of Individuals** | **Mean Length**  **(mm)** | **Mean Weight**  **(g)** | **Modal Sex** | **Modal Age** | **Modal Maturity** |
| --- | --- | --- | --- | --- | --- | --- | --- | --- | --- | --- |
| Mathair a'Gharbh Uilt | 1 | 8 | | Trout | 7 | 178.86 | 64.86 | F | 3+ | 2 |
|  | 2 | 5-6 | | Trout | 5 | 201.40 | 90.6 | F | 4+ | 3 |
| Loch a'Garbh-bhaid Mòr | 1 | 6-7 | | Trout | 12 | 163.58 | 67.58 | M | 3+ | 1 |
|  | 2 | 12-16 | | Charr | 16 | 139.56 | 36.38 | F | ***** | 3 |
|  | 3 | 25-32 | | -- | | | | | | |
| Loch na Tuadh | 1 | 15 | | -- | | | | | | |
|  | 2 | 3-5 | | Trout | 19 | 158 | 49.42 | F | 3+ | 1 |

*****Charr otolith age determination was unable to be conducted due to samples being misplaced.

**Table S4.** Captured Arctic charr (*S. alpinus*) NORDIC Multi Mesh gill net results for Loch a’Garbh-bhaid Mòr. [Date of Survey Night: 19/08/21]. 12 brown trout (*S. trutta*) were captured in Net 1 (6-7m depth) with no fish captured in Net 3 (25-32m depth). Maturity classification is based on Kesteven, (1960).

| **Net# (Depth/m)** | **Species** | **Length/mm** | **Weight/g** | **Sex** | **Maturity** | **Flesh #** |
| --- | --- | --- | --- | --- | --- | --- |
| N.2 (12-16) | Charr | 170 | 57 | m | 3 | 1 |
| N.2 (12-16) | Charr | 176 | 64 | m | 3 | 3 |
| N.2 (12-16) | Charr | 160 | 47 | f | 3 | 1 |
| N.2 (12-16) | Charr | 160 | 53 | f | 3 | 1 |
| N.2 (12-16) | Charr | 154 | 47 | m | 2 | 1 |
| N.2 (12-16) | Charr | 160 | 51 | f | 4 | 1 |
| N.2 (12-16) | Charr | 153 | 42 | m | 3 | 2 |
| N.2 (12-16)* | Charr | 145 | 37 | m | 2 | 1 |
| N.2 (12-16) | Charr | 149 | 45 | f | 2 | 1 |
| N.2 (12-16) | Charr | 149 | 40 | f | 2 | 1 |
| N.2 (12-16) | Charr | 123 | 22 | f | 2 | 1 |
| N.2 (12-16) | Charr | 113 | 18 | f | 1 | 1 |
| N.2 (12-16) | Charr | 111 | 17 | m | 1 | 1 |
| N.2 (12-16) | Charr | 110 | 17 | - | 1 | 1 |
| N.2 (12-16) | Charr | 95 | 11 | - | 1 | 1 |
| N.2 (12-16)* | Charr | 105 | 14 | - | 1 | 1 |

*No otolith collected


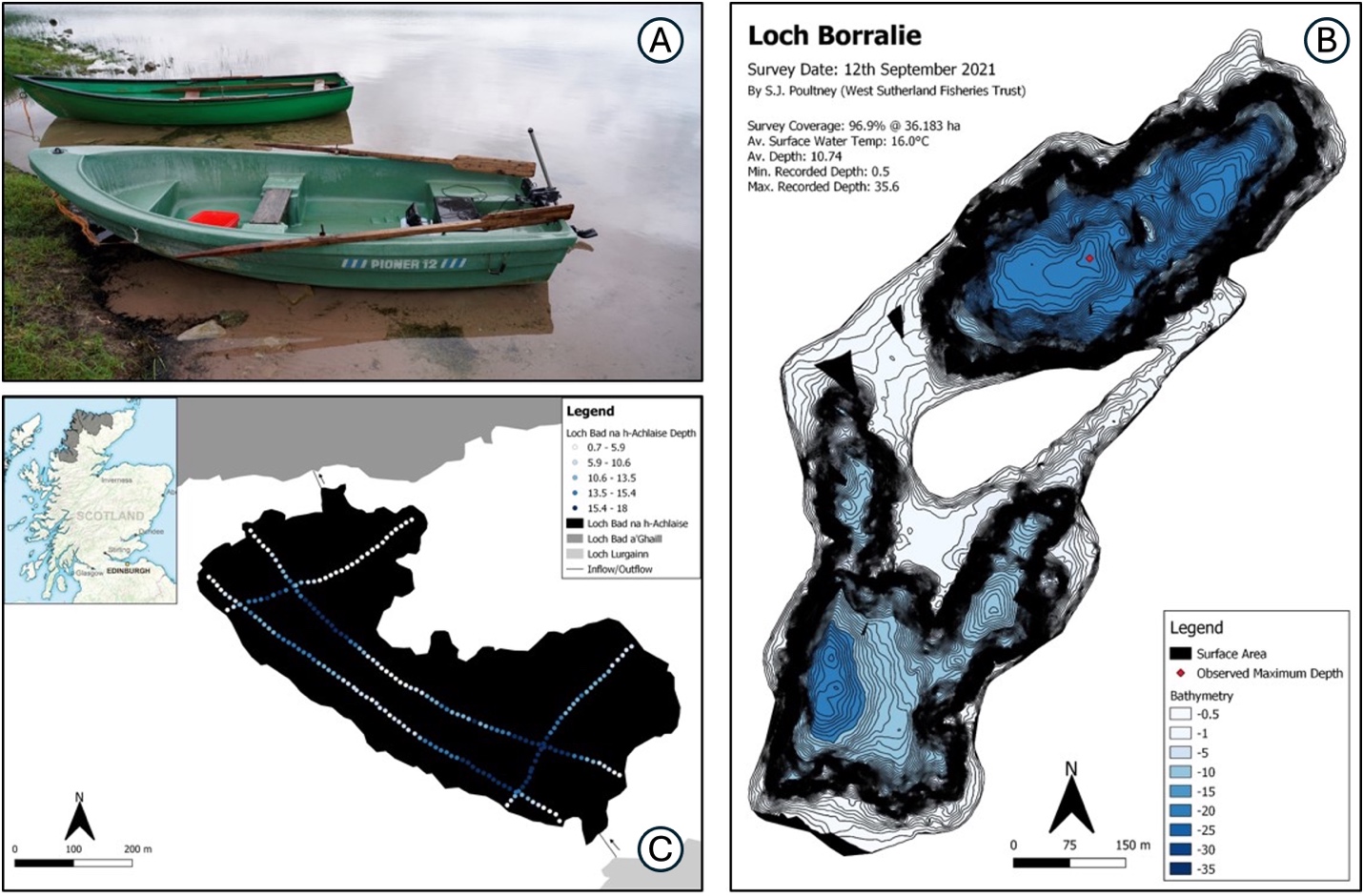


**Figure S1**: a) The Lowrance hydroacoustic system deployed on the transom of a Pioneer 12 rowing boat; b) a full hydroacoustic bathymetry survey result for Loch Borralie, highlighting 0.5m depth contours and other parameters; c) the result of a pack raft based bathymetric survey for Loch Bad an h-Achlaise;


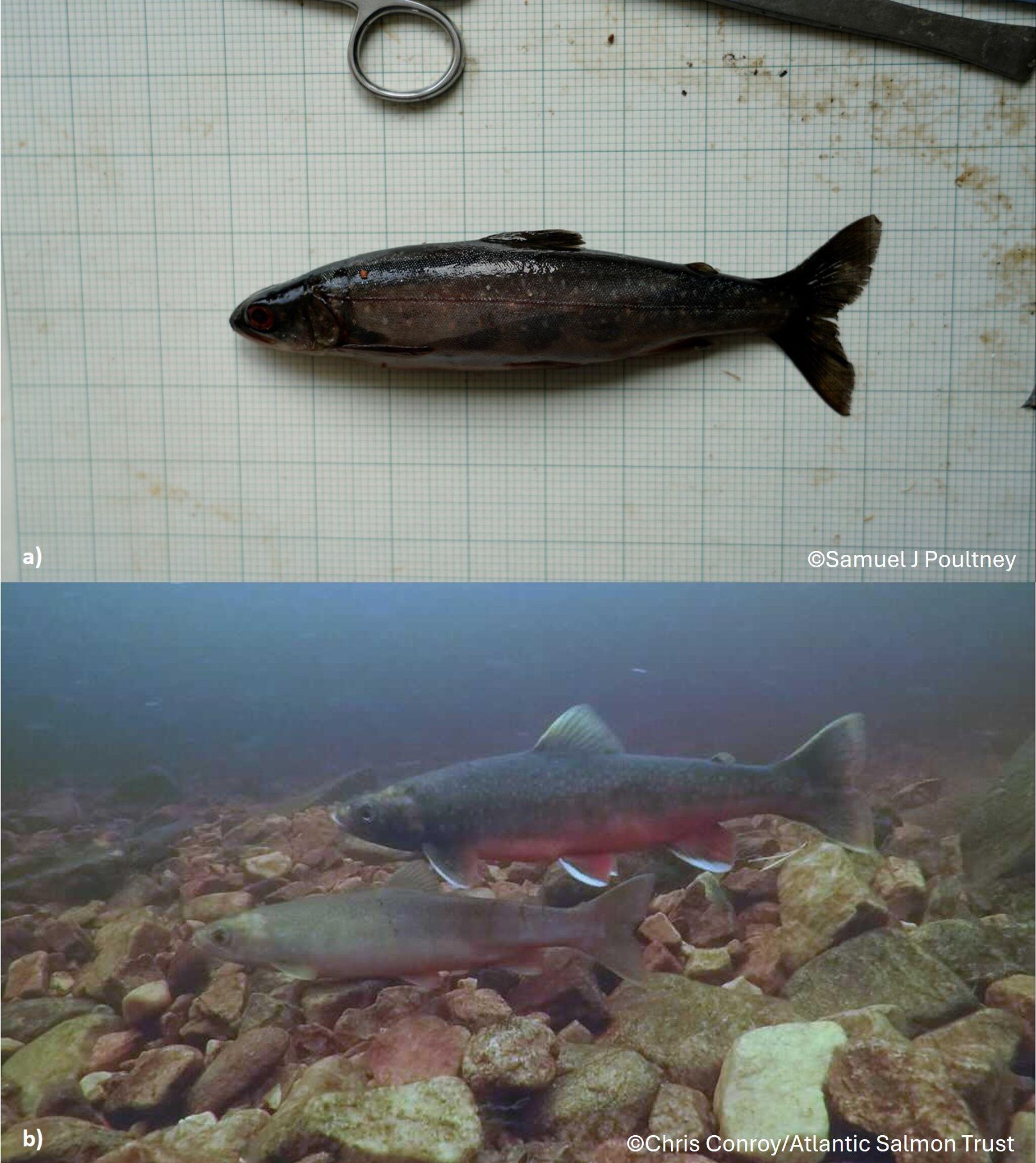


**Figure S2a**) Loch a’Garbh-bhaid Mòr specimen captured through NORDIC Net sampling; **2b**) and charr courting/spawning activity from Loch Stack inflow burns (Dec 2021). Courtesy: Chris Conroy/ Atlantic Salmon Trust

**Supplementary References**

Appelberg, M., Berger, H., Hesthagen, T., Kleiven, E., Kurkilahti, M., Raitaniemi, J. & Rask, M. (1995) Development and intercalibration of methods in Nordic freshwater fish monitoring. *Water, Air, and Soil Pollution*, **85**, 401-406

Bean, C. W. (2003) *A standardised survey and monitoring protocol for the assessment of Arctic charr, Salvelinus alpinus L., populations in the UK*. Final Report. Scottish Natural Heritage.

CEN (European Committee for Standardization), 2005: EN 14757. *Water quality – Sampling of fish with multi-mesh gill nets*. Brussels, 27 pp.

Kesteven, G. L. (Ed) (1960) Manual of field methods in fisheries biology. *F.A.O. Manuals in Fisheries Sciences*, No. 1, F.A.O. Rome. 152pp In Bagenal, T., (Ed) (1978) *Methods for Assessment of Fish Production in Fresh Waters*. 3 Ed, IBP Handbook: Oxford.

Miller, H., Winfield, I. J., Fletcher, J. M., Ben James, J., Rijn, J., Bull, J. M. & Cotterill, C.J. (2015) Distribution, characteristics and condition of Arctic charr (*Salvelinus alpinus*) spawning grounds in a differentially eutrophicated twin‐basin loch. *Ecology of Freshwater Fish,* **24**, 32-43

Murray, J. & Pullar, L. (1910) *Bathymetrical survey of the Scottish freshwater lochs*. Edinburgh: Challenger Office.

Winfield, I. J., van Rijn, J. & Valley, R.D. (2015) Hydroacoustic quantification and assessment of spawning grounds of a loch salmonid in a eutrophicated water body. *Ecological Informatics,* **30**, 235-240
